# The combined analysis as the best strategy for Dual RNA-Seq mapping

**DOI:** 10.1590/1678-4685-GMB-2019-0215

**Published:** 2020-02-10

**Authors:** Eliandro Espindula, Edilena Reis Sperb, Evelise Bach, Luciane Maria Pereira Passaglia

**Affiliations:** 1 Universidade Federal do Rio Grande do Sul (UFRGS), Instituto de Biociências, Departamento de Genética, Porto Alegre, RS, Brazil.

**Keywords:** Dual RNA-Seq, sequential analysis, combined analysis, mapping strategies

## Abstract

In Dual RNA-Seq experiments the simultaneous extraction of RNA and analysis of gene expression data from both interacting organisms could be a challenge. One alternative is separating the reads during *in silico* data analysis. There are two main mapping methods used: sequential and combined. Here we present a combined approach in which the libraries were aligned to a concatenated genome to sort the reads before mapping them to the respective annotated genomes. A comparison of this method with the sequential analysis was performed. Two RNA-Seq libraries available in public databases consisting of a eukaryotic (*Zea mays*) and a prokaryotic (*Herbaspirillum seropediceae*) organisms were mixed to simulate a Dual RNA-Seq experiment. Libraries from real Dual RNA-Seq experiments were also used. The sequential analysis consistently attributed more reads to the first reference genome used in the analysis (due to cross-mapping) than the combined approach. More importantly, the combined analysis resulted in lower numbers of cross-mapped reads. Our results highlight the necessity of combining the reference genomes to sort reads previously to the counting step to avoid losing information in Dual RNA-Seq experiments. Since most studies first map the RNA-Seq libraries to the eukaryotic genome, much prokaryotic information has probably been lost.

## Introduction

Organisms modulate their gene expression in order to establish many interactions, from pathogenic to beneficial relationships (Wolf *et al.*, 2018). There is a myriad of eukaryotic-prokaryotic interaction systems being studied, mainly focusing on pathogens and host gene expression responses, and pathogen-associated molecular patterns (PAMPs) (Westermann *et al.*, 2012). Besides that, another successful molecular interaction being widely studied is the relationship between plants and beneficial plant growth promoting bacteria (PGPB), which finds application in the understanding of agricultural inoculants (Balsanelli *et al.*, 2016, Bruto *et al.*, 2014, Camilios-Neto *et al.*, 2014).

Changes in gene expression or transcriptomes were first studied by microarray experiments focusing on only one of the interacting organisms (Barret *et al.*, 2009, Mela *et al.*, 2011). Recently, the RNA sequencing methodology (RNA-Seq) constitutes a promising approach for the parallel study of both interacting organisms, which was called Dual RNA-Seq (Westermann *et al.*, 2012). In the beginning, this technique presented some restrictions related to cost and a significant amount of data management, which is being surpassed by the advent of new sequencing methodologies and bioinformatic tools. However, many RNA-Seq experiments still focused in only one organism of the interaction (Hegedüs *et al.*, 2009, Boscari *et al.*, 2013, Pankievicz *et al.*, 2016, Verwaaijen *et al.*, 2017), whereas others assessed the transcriptome of both interacting organisms (Westermann *et al.*, 2012, Choi *et al*, 2014, Aprianto *et al.*, 2016, Westermann *et al.*, 2016, Reeder *et al.*, 2017, Westermann and Vogel 2018, Wolf *et al.*, 2018).

To perform a Dual RNA-Seq, steps of RNA isolation from both organisms, rRNA depletion, and cDNA library construction were adapted from the ones applied to simple RNA-Seq experiments (Westermann *et al.*, 2012). To analyze Dual RNA-Seq data, there are two approaches to choose: sequential or combined analysis (Wolf *et al.*, 2018). As the names say, the former consists of the sequential analysis of the libraries against the reference genomes, one after the other (Camilios-Neto *et al.*, 2014). In this approach, reads that fail to map to the first chosen reference genome are assumed to belong to the second genome. Therefore, these unmapped reads are the only ones used to map to the second genome (Packard *et al.*, 2017, Verwaaijen *et al.*, 2017). On the other hand, in combined analysis the libraries are aligned to a chimeric reference genome by concatenating the reference genomes (Aprianto *et al.*, 2016). All reads that aligned equally well to both genomes or have low alignment accuracy are removed.

Even though both methodologies described above are used to analyze Dual RNA-Seq data, they apparently are simple adaptations from the RNA-Seq methodologies that analyze one transcriptome at the time (Förstner *et al.*, 2014, Westermann *et al.*, 2016) and it seems critical to compare and evaluate which is the best choice for Dual RNA-Seq experiments. It is also worth considering that there is no consensus about the use or not of sequences that can align in more than one genome. Even though simultaneous read mapping has been suggested in 2012 (Westermann *et al.*, 2012), most of the Dual RNA-Seq works still opt to use the sequential approach (Kovalchuk *et al.*, 2019; LaMonte *et al.*, 2019; Mateus *et al.*, 2019; Montoya *et al.*, 2019; Mutha *et al.*, 2019).

Here we present a mapping strategy for the combined analysis that consists of: i) aligning the Dual RNA-Seq libraries against a single file containing both reference genomes; ii) after this first mapping procedure, the reads attributed to each genome are extracted and saved into separated files; iii) these files are then used as individual libraries for the counting step using the respective annotated genome. Besides that, we present comparisons of this methodology to the sequential analysis to emphasize the importance of carefully choosing the mapping strategies for Dual RNA-Seq analysis. We test our approach using RNA-Seq libraries from different interaction systems. In two of them, we used data available in public databases, whereas in another analysis, the RNA-Seq libraries were part of an experiment performed in our laboratory that aimed to study the interaction between *Glycine max* roots and the bacterium *Bradyrhizobium elkanii.*

## Material and Methods

### RNA-Seq libraries and reference genomes

In order to test the combined analysis, we used RNA-Seq libraries available in public databases.

Firstly, data from two independent works were used to simulate a Dual RNA-Seq library. These data consisted in: NT-1 and NT-2 libraries from the bacterium *Herbaspirillum seropedicae* SmR1, available in the ArrayExpress database under the accession number E-MTAB-2842 (Bonato *et al.*, 2016); and four mRNA libraries isolated from the central portion of the starchy endosperm of *Zea mays* (maize) cv. B73 six days after pollination, available in the NCBI database under the accession number SRP043224 (Thakare *et al.*, 2014). *Herbaspirillum* and maize libraries were merged into a single file for each organism.

To verify if the results observed using the individual (and the Chimera) libraries are repeated in real Dual RNA–seq experiments, libraries from two Dual RNA–seq experiments were also evaluated. The first dataset was comprised of dual RNA-Seq paired-end data from Lanubile *et al.* (2014), who investigated maize root genes involved in the defensive response to the infection caused by the fungus *Fusarium verticillioides*. Libraries of the biological replicates of the susceptible maize variety CO354 inoculated with *F. verticillioides* were obtained from NCBI, accession numbers SRR1186869, SRR1186870, and SRR1186871 (Lanubile *et al.*, 2014).

The second dataset was obtained from an unpublished experiment performed in our laboratory that consisted of Dual RNA-Seq single-end libraries. The experiment was designed to evaluate the interaction between two varieties of soybean (*Glycine max*, EMBRAPA 48 and BR 16) with the bacterium *Bradyrhizobium elkanii* strain SEMIA 587. Libraries were obtained as described below and were deposited at NCBI under the accession numbers: SRR7206486: BR16, replicate I; SRR7206485: BR16, replicate II; SRR7206490: EMBRAPA 48, replicate I; SRR7206489: EMBRAPA 48, replicate II.

Reference genomes of *B. elkanii* USDA76 (GCF_000379145.1), *G. max* (GCF_000004515.5), *H. seropedicae* Z67 (GCF_001040945.1), *Fusarium verticillioides* (GCF_000149555.1) and *Z. mays* cv. B73 (GCF_000005015.2) and their respective annotations were obtained from NCBI.

### Data analysis

The CLC Genomics Workbench 8.0 (CLC – Bio; QIAGEN) toolkit was used to perform the trimming, mapping, and counting steps. The “Trimming” tool was used to trim reads smaller than 20 nucleotides from the RNA-Seq libraries, according to the program default settings for quality control. The “Convert to Tracks” tool was applied to the reference genomes to correctly associate them to the respective annotations.

Trimmed *H. seropedicae* and *Z. mays* RNA-Seq libraries were aligned to their respective reference genomes to eliminate possible contaminant reads, using the “Map to a Reference” tool with the parameters set to 0.8 of minimum length fraction and 0.8 of minimum similarity fraction. This procedure was called direct mapping and the libraries were called filtered libraries (Figure S1A). Both filtered libraries (from *Herbaspirillum* or maize) were exported as separate fastq files, which were further merged into a single file to form a Chimera Library to simulate a Dual-RNA-Seq experiment (Figure S1B).

We considered cross-mappings the number of reads that belonged to one organism’s transcriptome that mapped to the other organism’s genome. To check for cross-mapping, each RNA-Seq filtered library was aligned to the reference genome of the other organism (Figure S2A). Both cross-mapping and contamination checking steps were useful to further evaluate our results. The *H. seropedicae* and *Z. mays* reference genomes were also merged into a single file (Combined Reference), and each RNA-Seq filtered library from *Herbaspirillum* and maize was aligned to the Combined Reference file (Figure S2B).

**Figure 1:**
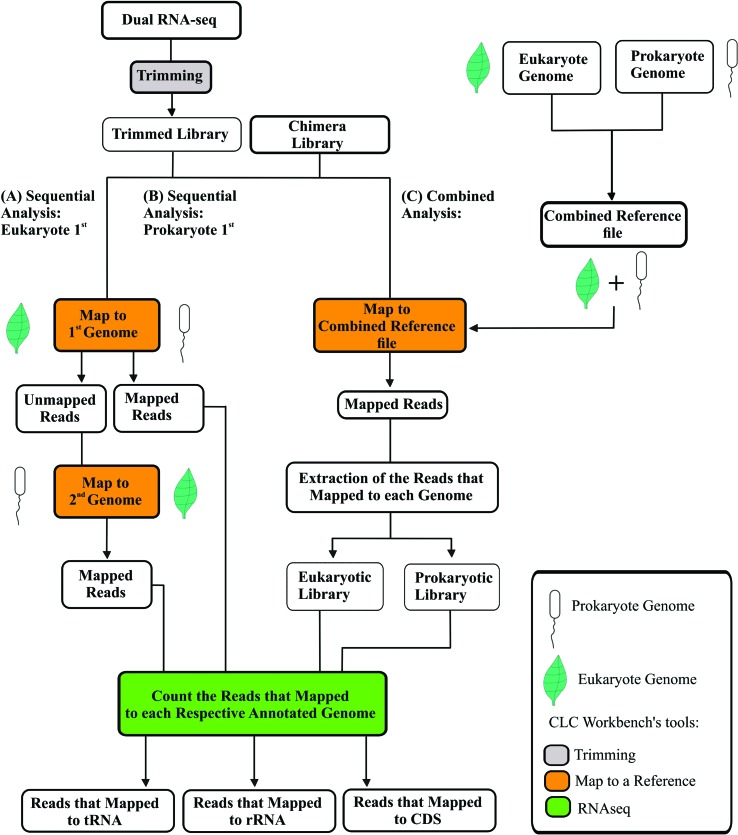
Mapping strategies for Dual RNA-Seq analysis. (A) Sequential analysis aligning libraries to the eukaryotic genome first- Eukaryote 1^st^; (B) Sequential analysis aligning libraries to the prokaryotic genome first- Prokaryote 1^st^; (C) Combined analysis.

The Chimera Library was used for the sequential and combined analyses and mapping was done with the “Map to a reference” tool of CLC’s program. The first sequential analysis was performed aligning reads to the maize reference genome to generate the first set of data (Eukaryote first- Figure 1A). Afterward, the exact opposite was performed, and the reads were mapped against the bacterium reference genome first to produce the second set of data (Prokaryote first- Figure 1B). In the combined analysis, we aligned the Chimera Library to the Combined Reference file to sort out the sequences belonging to one or another genome (Figure 1C). This Combined Reference was made by concatenating the files of the maize and *Herbaspirillum* reference genomes into a single reference file. For this purpose, we used the command "cat" of the Linux terminal to merge files. After sorting the sequences, those attributed to each genome were extracted and exported as separate fastq files. Files were imported back to CLC to count the reads of each library as described below.

Reads from RNA-Seq libraries of *Z. mays*, *H. seropedicae,* and from the Chimera library that aligned to tRNA, rRNA, and to CDS (coding DNA sequence) loci were counted using the CLC’s tool “RNAseq” with the parameters set to 0.8 of minimum length fraction and 0.8 of minimum similarity fraction, not mapping to intergenic regions, and allowing a maximum of 5 hits (Figure 1).

To verify if a more rigorous set up condition could improve the results, the analyses were also done using the parameters of 0.9 of minimum length fraction and 0.8 of minimum similarity fraction. This condition is most commonly used for bacterial RNA-seq library alignments (Camilios-Neto *et al.*, 2014, Bonato *et al.*, 2016).

The specificity, sensitivity, accuracy, and precision of each mapping method were also calculated, estimating the true positives, the true negatives, the false positives, and the false negatives reads of each condition. Table S1 details which reads were considered in each group.

In order to compare the results observed for the Chimera Library, the dual RNA-Seq libraries obtained in the soybean/*Bradyrhizobium* and maize/*Fusarium* experiments (Lanubile *et al.*, 2014) were also analyzed using the sequential and combined approaches with the parameters of 0.8 of minimum length fraction and 0.8 of minimum similarity fraction. In the maize/*Fusarium* experiment, some reads were mapped as broken pairs. Although these reads could align independently, none of the possible placements of the pair satisfied the pairing criteria. These reads were then treated as independent and marked as broken pairs. As these reads satisfied the mapping criteria, they were maintained in the following steps of the analysis.

### Soybean varieties, bacterial strain, inoculation, growth, and experimental conditions

Soybean (*Glycine max)* plants of the contrasting genotypes EMBRAPA 48 and BR 16 (Oya *et al.*, 2004) were grown under controlled temperature (26 ± 4 ºC), luminosity (~ 600 μmol m^-2^ s^-1^), and photoperiod (18/6 h light/dark). Cultivation was carried out in magenta boxes sealed in the root system, under a hydroponic system. Nutrients were supplied through the Hoagland’s nutritive solution strength (Hoagland and Arnon, 1938), which was replaced every three days. The nutritive solution was modified lacking nitrogen to stimulate nodulation. Soybean seeds were surface-sterilized by washing them three times with autoclaved ultrapure water, followed by soaking them in 70% ethanol for 3 min, and by a solution of 2% sodium hypochlorite and 2.5% Tween 20 for 30 min. Seeds were then washed three times with sterile distilled water by gentle shaking (Faleiro *et al.*, 2013). All solutions and materials used were sterilized at 120 ºC for 30 min. When the V2-V3 stage (Fehr *et al.*, 1971) was reached, seedlings were inoculated with the symbiotic bacterium *Bradyrhizobium elkanii* SEMIA 587. *B. elkanii* was cultivated in yeast-mannitol liquid medium (Somasegaran and Hoben, 1994) in an orbital shaker (28 °C, 120 rpm). When cultures reached an OD_600_ of 0.6, they were collected and centrifuged for 10 min at 10,000 x *g* at 4 °C. The resulting pellets were washed twice with sterile 0.85% NaCl solution, suspended in the same solution, and then diluted to obtain the inoculation solution at a concentration of approximately 10^8^ CFU/mL (colony forming units).

Inoculation of the roots was performed by submerging them into the inoculant solution for 60 s. Inoculated roots were immediately frozen in liquid nitrogen and cryopreserved at -80 °C for subsequent RNA isolation. Two biological replicates composed of pooled root seedlings from five plants were used for each genotype, resulting in four composed samples for further RNA isolation.

### RNA isolation, mRNA enrichment, cDNA synthesis, and sequencing

Total RNA isolation of *G. max* root seedlings inoculated with *B. elkanii* was done using TRIzol (Invitrogen) reagent. The integrity of RNA was verified on 1.5% agarose gel. Concentration and purity were determined by spectrophotometry at 260 nm and 280 nm (Jahn *et al.*, 2008) measured in Nanodrop LITE spectrophotometer (Thermo Fisher Scientific). RNA samples were subjected to a purification step using PureLink RNA Micro kit (Ambion), treated with DNaseI (Invitrogen) and then rRNA was depleted using the RiboMinus Plant Kit for RNA-Seq (Invitrogen). The cDNA libraries were constructed using the Ion total RNA-Seq kit v2 for Whole Transcriptome Library. All RNA quantification and quality evaluation were performed at the Bioanalyzer - Agilent 2100 instrument. Each cDNA library obtained was sequenced using the Ion PI Template OT2 200 Kit v3 and the Ion PI Sequencing 200 Kit v3 at the IonTorrent® platform (Thermo Fisher Scientific). All kits and reagents were used according to manufacturer’s instructions.

The presence of the bacterium in plant roots was subsequently determined by the detection of its 16S rRNA gene sequences in the transcriptome library.

## Results

### Data analysis using independent RNA-Seq libraries

Before starting the analysis, trimmed RNA-Seq libraries from *Z. mays* and *H. seropedicae* were filtered by direct mapping to each genome to avoid potential contamination sequences (Figure S1A). After filtering, the *H. seropedicae* RNA-Seq library presented approximately 44 million reads, while the *Z. mays* RNA-Seq library presented approximately 22 million reads. The Chimera Library, which simulates a Dual RNA-Seq experiment, was constructed joining these two libraries (Figure S1B) and presented approximately 66 million reads (Table 1).

**Table 1:** Library features and number of total reads attributed to the *Herbaspirillum seropedicae* or *Zea mays* genomes according to the mapping approach. The analyses were performed with the genomes without annotations, with the mapping parameters of 0.8 of minimum length fraction and 0.8 of minimum similarity fraction. Values for sensitivity, specificity, accuracy, and precision were determined according to Table S1.

Library	Total Reads	Total reads after trimming	Number of Reads After Library filtration	Cross-Mapping^1^		Mapping Strategy						
					Sequential Analysis^2^					Combined Analysis^3^		
					Eukaryote 1st		Prokaryote 1st				
				*H. seropedicae*	*Z. mays*	*H. seropedicae*	*Z. mays*	*H. seropedicae*	*Z. mays*	*H. seropedicae*	*Z. mays*	unmapped
*H. seropedicae*	158,053,843	92,987,843	44,469,308	-	13,847,693	-	-	-	-	43,661,668	779,556	28,084
*Z. mays*	24,300,211	24,255,170	22,200,875	7,659	-	-	-	-	-	394	22,200,465	16
*Chimera Library*	-	-	66,670,183	-	-	30,621,615	36,048,568	44,476,967	22,193,216	43,662,066	22,980,017	28,100
Sensitivity	-	-	-	-	-	0.6886	1.0000	1.0000	0.9997	0.9825	1.0000	-
Specificity	-	-	-	-	-	1.0000	0.6886	0.9997	1.0000	1.0000	0.9825	-
Accuracy	-	-	-	-	-	0.7923	0.7923	0.9999	0.9999	0.9883	0.9883	-
Precision	-	-	-	-	-	1.0000	0.6159	0.9998	1.0000	1.0000	0.9661	-

1 Cross-mapping: reads of each individual library were mapped to the reference genome of the other organism.

2 Sequential Analysis: The library was first mapped to one reference genome, reads that fail to map to the first genome were mapped to the other genome. Eukaryote 1^st^/Prokaryote 1^st^ indicates the first reference used.

3 Combined analysis: libraries were mapped to a merged file containing both reference genomes (Combined Reference).

Cross-mappings were determined by the number of reads from one organism RNA-Seq library that could be attributed to the other organism reference genome (Figure S2A). Interestingly, approximately 13 million reads from the *H. seropedicae* RNA-Seq library aligned to the *Z. mays* genome, while 7,659 reads from the *Z. mays* RNA-Seq library mapped to the *H. seropedicae* genome (Table 1). On the other hand, when we mapped the individual RNA-Seq libraries to the Combined Reference file (Figure S2B), which was constructed by concatenating *H. seropedicae* and *Z. mays* genomes (Figure 1), more surprising results were obtained. When the *H. seropedicae* RNA-Seq library was aligned to the Combined Reference file, approximately 43 million reads were attributed to *H. seropedicae* genome and 779,556 reads to *Z. mays* genome; whereas when *Z. mays* RNA-Seq library was mapped to the Combined Reference file, 394 reads were attributed to *H. seropedicae* genome and approximately 22 million reads to *Z. mays* genome (Table 1). These results showed that even in the presence of both reference genomes some reads still mapped incorrectly, although the numbers of reads incorrectly aligned were much smaller than the numbers of cross-mapping reads obtained when one RNA-Seq library was aligned to the genome of the other organism.

After estimating cross-mappings, we evaluated both the sequential and the combined approach of Dual RNA-Seq analysis. The sequential analysis consisted of aligning the Chimera Library to one reference genome before the other. Reads that aligned to the first genome constituted this organism’s library. Unmapped reads are then mapped to the second reference genome. All reads that aligned to the second genome comprised this organism’s library. We first mapped the Chimera Library to *Z. mays* reference genome, and then the unmapped reads were mapped to the *H. seropedicae* reference genome (Figure 1A). Approximately 30 million reads were attributed to *H. seropedicae* genome, and approximately 36 million reads were attributed to *Z. mays* genome (Table 1 - Eukaryote 1^st^). When we did the opposite and first mapped the Chimera Library to the *H. seropedicae* reference genome (Figure 1B), approximately 44 million reads were attributed to *H. seropedicae* genome and approximately 22 million reads to *Z. mays* genome (Table 1 – Prokaryote 1^st^).

Finally, we performed the combined analysis that consists of aligning the RNA-Seq library to a file containing a combination of reference genomes (Combined Reference). We mapped the Chimera Library to the Combined Reference file, and this alignment approach attributed approximately 43.6 million reads to *H. seropedicae* genome and approximately 22.9 million reads to *Z. mays* genome (Table 1 – Combined analysis, Figure 1C). After the mapping procedure, reads attributed to each genome were extracted, saved into separated files (Figure S3), and used as individual libraries for the counting step (Figure 1C). All reads that aligned to *H. seropedicae* or *Z. mays* genomes were counted using the corresponding reference genome and its respective annotations (Tables 2 and 3). In all counts we observed unmapped reads. This is likely due to the parameters chosen for counting the reads in the CLC’s “RNAseq” tool, as reads that mapped in more than five loci or mapped in intergenic regions were excluded.

**Table 2:** Number of reads mapped to tRNA, rRNA, and coding loci (CDS) according to the mapping methodology used, with the mapping parameters of 0.8 of minimum length fraction and 0.8 of minimum similarity fraction.

Mapping strategy	Library	Reference used to count the reads	Number of reads mapped to				Unmapped reads	Proportion of multi-reads from total
			tRNA	rRNA	CDS loci			
					Unique	Multi		
Direct Mapping	*H. seropedicae*	*H. seropedicae*	1,423,990	31,630,385	9,005,409	79,429	2,330,095	0.18%
	*Z. mays*	*Z. mays*	1,692	3,003	20,163,387	888,259	1,144,534	4.00%
Eukaryote 1st	Chimera Library	*H. seropedicae*	1,181,068	21,550,448	6,052,838	31,666	1,805,595	0.10%
		*Z. mays*	89,216	3,254,994	24,843,247	2,591,042	5,270,069	7.19%
Prokaryote 1st	Chimera Library	*H. seropedicae*	1,423,992	31,631,168	9,010,911	80,116	2,330,780	0.18%
		*Z. mays*	1,686	2,255	20,157,930	887,162	1,144,183	4.00%
Combined Analysis	Chimera Library	*H. seropedicae*	1,419,674	31,304,115	8,591,366	59,339	2,287,572	0.14%
		*Z. mays*	1,971	79,917	20,530,853	1,052,003	1,315,273	4.58%

**Table 3:** Comparison of the number of reads incorrectly mapped due to cross-mapping, with the mapping parameters of 0.8 of minimum length fraction and 0.8 of minimum similarity fraction. Reads that incorrectly mapped to the reference genome were counted using the annotated genome indicated on the table. The unmapped reads are a result of the counting parameters that eliminate reads that mapped in more than five loci and of the intergenic regions.

Library	Reference Used to Map the Reads	Reference Used to Count the Cross-Mapped Reads	Number of Reads Mapped to			CDS*	Unmapped reads
			tRNA	rRNA	CDS Loci		
*H. seropedicae*	*Z. mays*	*H. seropedicae*	242,922	10,079,937	3,000,334	4,553	524,500
		*Z. mays*	87,524	3,251,991	6,382,643	23,216	4,125,535
	Combined Reference	*H. seropedicae*	4,156	320,514	413,822	3,299	41,064
		*Z. mays*	279	77,231	531,276	3,298	170,770
*Z. mays*	*H. seropedicae*	*Z. mays*	6	748	6,554	72	351
		*H. seropedicae*	2	783	6,189	65	685
	Combined Reference	*Z. mays*	0	308	57	43	29
		*H. seropedicae*	0	329	59	49	6

*CDS with at least 10 reads assigned to them. Exception made to the *Z. mays* library mapped against the Combined Reference, which refers to CDS with at least one read assigned to it.

The counting of reads that aligned to the respective genome in the direct, sequential, and combined analysis showed interesting results (Table 2). In the direct analysis, for the *H. seropedicae* RNA-Seq library, 1,423,990 reads were attributed to tRNA, 31,630,385 reads to rRNA, and 9,00,409 (unique mapped) reads to CDS loci using the *H. seropedicae* genome, 616,765 remained unmapped*,* while for *Z. mays* RNA-Seq library, we counted 1,692 tRNA reads, 3,003 rRNA reads, and 21,051,646 CDS loci reads using the *Z. mays* genome, 1,144,534 remained unmapped (Table 2 – Direct mapping). In the sequential analysis, when we first mapped the Chimera Library to the *Z. mays* genome, we counted using the genome of *H. seropedicae* 1,181,068 tRNA reads, 21,550,448 rRNA reads, 6,052,838 (unique mapped) CDS loci reads, and 1,805,595 reads remained unmapped*,* while when using the genome of *Z. mays*, we counted 89,216 tRNA reads, 3,254,994 rRNA, 24,843,247 (unique mapped) CDS loci reads, and 5,270,069 reads remained unmapped (Table 2 – Eukaryote 1^st^). On the other hand, when we first mapped the Chimera Library to the *H. seropedicae* genome and also counted with *H. seropedicae* files, 1,423,992 reads were attributed to tRNA, 31,631,168 reads to rRNA, 9,010,911 (unique mapped) to CDS loci, and 2,330,780 reads remained unmapped*,* while when counting using the *Z. mays* genome, 1,686 reads were attributed to tRNA, 2,255 reads to rRNA, 20,157,930 (unique mapped) reads to CDS loci, and 1,144,183 reads remained unmapped (Table 2 – Prokaryote 1^st^). Finally, when we counted the reads that mapped to each reference genome using the combined analysis and counted using *H. seropedicae* files, 1,419,674 reads were attributed to tRNA, 31,304,115 reads to rRNA, 8,591,366 (unique mapped) reads to CDS loci, and 2,287,572 reads remained unmapped*,* while when counting with the *Z. mays* genome, 1,971 reads were attributed to tRNA, 79,917 reads to rRNA, 20,530,853 (unique mapped) reads to CDS loci, and 1,315,273 reads remained unmapped (Table 2 – Combined analysis).

The amount of multi–mapped reads assigned to CDS loci was also evaluated. We observed that at least 4% of the reads attributed to maize CDS are multi–mapped reads. For *Herbaspirillum* this amount corresponded to less than 1% of the total reads attributed to *Herbaspirillum* CDS (Table 2).

All the analyses described above were also performed using 0.9 of minimum length fraction and 0.8 of minimum similarity fraction as a more stringent parameter. After the filtering step, the *H. seropedicae* RNA-Seq library presented approximately 41 million reads, while the *Z. mays* RNA-Seq library presented approximately 21 million reads. The Chimera Library presented approximately 62 million reads (Table S2). Comparing with the amount that was mapped to *Herbaspirillum* genome using the previous set up parameters, the amount of reads mapped to the *Herbaspirillum* genome was reduced in 3,137,800. After that, we determined the amount of reads that could cross-map. We highlight the fact that approximately 8.9 million reads from *Herbaspirillum* mapped to the *Zea mays* genome (Table S2), which represents a reduction of around 10% in the number of cross mappings reads. This reduction was probably caused by the reduction of mapped reads observed in the direct mapping. In the counting step we highlight the fact that there was a reduction in the number of identified CDS loci in almost all situations (data not show). For the remaining results, the observed patterns were the same of those observed for the previous set up (Table S3).

In order to investigate the reads that were incorrectly aligned (cross-mapped reads), those reads were also counted using both the respective and the incorrect reference genomes (Figure S2, Tables 3 and S4). When the parameters of 0.8 of minimum length fraction and 0.8 of minimum similarity fraction were applied, although several reads incorrectly mapped to rRNA and tRNA loci, the most important result was that about six million *H. seropedicae* reads were incorrectly attributed to almost 23,216 *Z. mays* CDS. A similar situation, although with minor effects, was observed for the *Z. mays* library, where 6,189 reads were incorrectly attributed to 65 *H. seropedicae* CDS. In these cases, only CDS that received at least ten reads assigned to them were considered. When the combined reference file was used, the numbers of reads incorrectly mapped decreased significantly, in particular for the *H. seropedicae* genome. In this case, 531,276 reads from *H. seropedicae* were incorrectly attributed to 3,298 *Z. mays* CDS. These analyses were also performed using the parameter of 0.9 of minimum length fraction and 0.8 of minimum similarity fraction. For these analyses all the results presented the same pattern observed for the previous parameter, in which the reads that cross mapped were mostly assigned to rRNA and CDS loci (Table S4).

We also estimated the sensitivity, specificity, precision, and accuracy (Table S1) of the methodologies for all parameters tested (Tables 1 and S2). Regardless of the parameter used, we observed that in the sequential analysis – Eukaryote 1^st^ the accuracy was lower than in the sequential analysis – Prokaryote 1^st^ or in the Combined Analysis (Tables 1 and S2). When comparing the values of these parameters for the sequential analysis – Prokaryote 1^st^ with those obtained for the Combined analysis, we observed that the accuracy values in both methodologies were equivalent for both parameters, with a slight increase when the more rigorous parameter was used. Taking all this together and to the fact that the mapping parameter of 0.9 of minimum length fraction and 0.8 of minimum similarity fraction lead to a reduction in the number of CDS loci identified (probably caused by the reduction of the amount of reads mapped in the direct mapping), the Combined Analysis with the parameters of 0.8 of minimum length fraction and 0.8 of minimum similarity fraction was used in the following analyses.

Tables S5 and S6 present the top 20 most counted loci among the cross-mapped reads. Table S5 shows the loci where the reads should be aligned in the correct genome, while Table S6 shows the loci where the reads aligned in the incorrect genome. It is interesting to note that most of the incorrectly mapped reads corresponded to genes that code for proteins with different functions, such as kinases, phosphatases, and ribosomal proteins. Several genes coding for hypothetical or uncharacterized proteins were also identified.

### Analyses of experimentally obtained Dual RNA-Seq libraries

**Figure 2:**
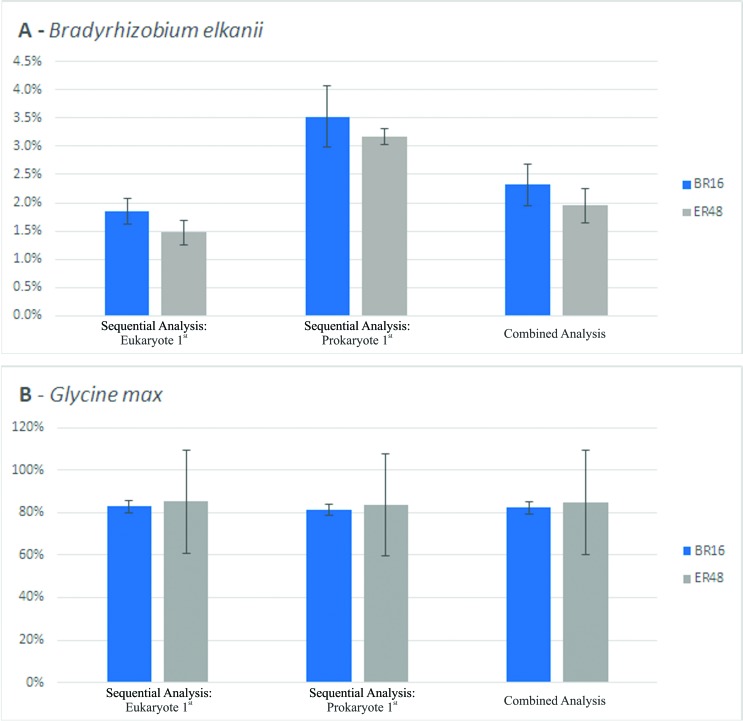
Percentage of reads mapped to (A) *Bradyrhizobium elkanii* or (B) *Glycine max* depending on the methodology used in the *Glycine max – Bradyrhizobium elkanii* experiment. Bars indicate twice the Standard Error. BR16 and ER48: soybean varieties BR16 and Embrapa 48, respectively.

The combined Dual RNA-Seq analysis was also applied to RNA-Seq libraries obtained from two experiments, one performed in our laboratory and another carried out by Lanubile *et al.* (2014). The first one aimed to evaluate the interaction of two varieties of *G. max* with the symbiotic bacterium *B. elkanii.* The presence of the bacterium in the plant’s roots was confirmed by the detection of its 16S rRNA gene sequences in the RNA-Seq libraries (data not shown). RNA-Seq libraries obtained from both organisms showed enough quality and coverage to perform gene expression analysis (Table S7). After the trimming procedure, RNA-Seq libraries from the *G. max - B. elkanii* experiment presented approximately 5 to 9 million reads (Table S7). The Dual RNA-Seq alignment strategies showed that numbers attributed to the eukaryotic genome roughly did not vary among sequential or combined analyses, regardless of the soybean variety used (Figure 2A, Table S7). However, some variation was observed in the number of reads mapped to the prokaryotic genome depending on the mapping approach.

When RNA-Seq reads were first aligned to the eukaryotic genome (Table S7 - Sequential analysis- Eukaryote 1^st^), the number of reads attributed to the bacterium was less than 2% of the total amount of reads (Figure 2B) for both soybean varieties. However, when the opposite analysis was performed (Table S7 - Sequential analysis- Prokaryote 1^st^), the number of reads aligned to the prokaryote genome increased significantly, reaching more than 3% of the total amount of reads mapped in both samples (Figure 2B), also for both soybean varieties. Using the combined analysis, when reads were aligned to both genomes at the same time, intermediary numbers of reads were attributed to the prokaryote genome, regardless of the soybean variety used (Table S7 – Combined analysis, Figure 2B). The average number of reads attributed to the prokaryote in the combined analysis was not significantly different from the average number attributed at the Sequential Analysis – Eukaryote 1^st^. Despite this fact, the results still indicated that probably some reads that mapped to the first genome used in the sequential approach very likely belong to the second genome and incorrectly mapped to the first genome because the second was not present in the analysis.

**Figure 3:**
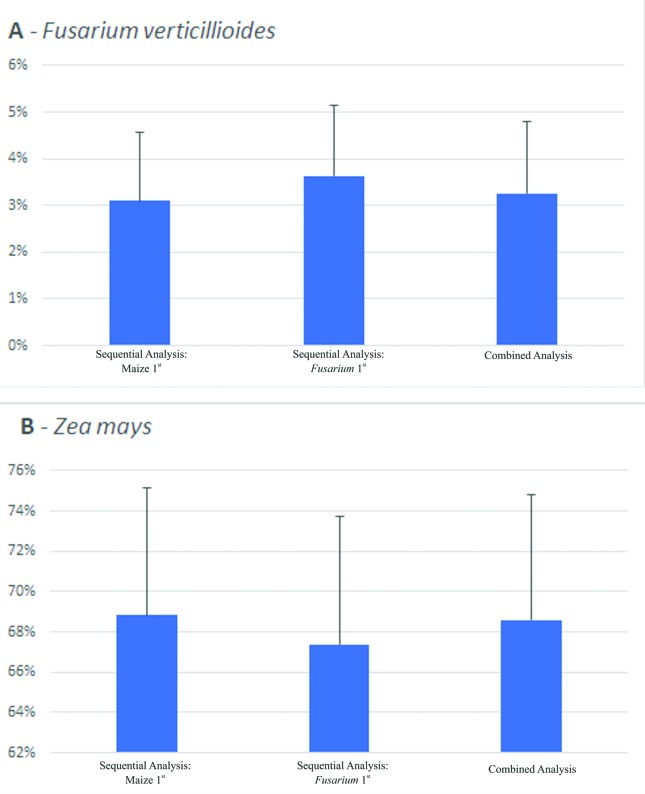
Percentage of reads mapped to (A) *Fusarium verticillioides* or (B) *Zea mays* depending on the methodology used in the *Zea mays –Fusarium verticillioides* experiment. Bars indicate twice the Standard Error.

The second experiment used to evaluate the combined Dual RNA-Seq analysis was performed by Lanubile *et al.* (2014), who investigated maize roots gene expression during *Fusarium verticillioides* infection. Although these authors investigated *Z. mays* genes only, library preparation involves the isolation of mRNAs using poly(A)-tails, which potentially included fungus mRNA. Thus, we chose this library as another example of plant-microorganism interaction. After the trimming procedure, the libraries had from 74 to 83 million reads (Table S8). In this analysis, even though the numbers of reads attributed to both genomes varied according to the previous experiments (Table S8), the average number of reads attributed to each genome according to the methodology used were not significantly different, since the standard errors were substantial (Figure 3). Nevertheless, the combined analysis showed an intermediate amount of reads attributed to each genome in comparison with the number of reads observed in the sequential analyses (Table S8, Figure 3), which was similar to the previous analyses.

We also evaluated the amount of multi–mapped reads attributed to CDS loci in both experiments. For the *Bradyrhizobium*–*Glycine max* experiment, we observed that more than 60% of the reads attributed to *G. max* CDS were multi–mapped reads. For the *Bradyrhizobium* we observed that less than 1% of the reads attributed to CDS loci were multi–mapped reads. In the *Fusarium*–maize experiment around 7% of the reads attributed to CDS loci in both organisms were multi–mapped reads (Table 4).

**Table 4:** Number of reads mapped to tRNA, rRNA, and coding loci (CDS) according to the mapping methodology, with the mapping parameters of 0.8 of minimum length fraction and 0.8 of minimum similarity fraction, and experiment used. BR16 and ER48: soybean varieties BR16 and Embrapa 48, respectively. CO354: susceptible maize variety CO354 inoculated with *F. verticillioides* from Lanubile *et al.* (2014).

Samples	Biological Repetition	Mapping Strategy	Reference Used to Count the Reads	Number of Reads Mapped to				Unmapped reads	Proportion of Multireads from total
				tRNA	rRNA	CDS loci			
*Soybean + Bradyrhizobium:*						Unique	Multi		
BR16	I	Eukaryote 1^st^	*G. max*	9,262	458,414	1,386,492	5,505,200	275,078	72.11%
	II		*G. max*	14,526	524,794	1,553,300	2,218,202	298,956	48.12%
	I		*B. elkanii*	6,140	137,368	18,423	408	1,349	0.25%
	II		*B. elkanii*	8,431	153,677	20,804	318	2,025	0.17%
ER48	I		*G. max*	7,486	284,116	853,250	34,198,215	161,811	96.32%
	II		*G. max*	12,566	400,613	1,496,084	4,115,974	263,977	65.44%
	I		*B. elkanii*	5,423	87,283	8,217	194	1,057	0.19%
	II		*B. elkanii*	5,219	64,293	16,609	524	1,401	0.60%
BR16	I	Prokaryote 1^st^	*G. max*	7,784	337,378	1,381,270	5,493,766	271,697	73.33%
	II		*G. max*	11,509	383,520	1,547,288	5,502,769	293,615	71.11%
	I		*B. elkanii*	8,597	262,024	30,995	919	3,704	0.30%
	II		*B. elkanii*	13,152	301,072	34,724	1,183	6,201	0.33%
ER48	I		*G. max*	5,803	209,395	848,055	3,406,326	158,528	73.60%
	II		*G. max*	10,145	321,792	1,485,462	4,091,851	258,616	66.34%
	I		*B. elkanii*	8,443	165,541	20,871	703	4,387	0.35%
	II		*B. elkanii*	9,329	147,323	43,393	2,016	7,363	0.96%
BR16	I	Combined Analysis	*G. max*	8,571	420,015	1,384,623	5,504,514	273,168	72.51%
	II	*G. max*	13,227	477,056	1,550,710	5,517,511	295,728	70.25%
	I		*B. elkanii*	7,097	172,756	18,760	493	1,595	0.25%
	II		*B. elkanii*	10,299	200,206	21,413	429	2,236	0.18%
ER48	I		*G. max*	6,617	252,998	850,854	3,418,620	159,801	72.91%
	II		*G. max*	11,597	378,032	1,493,400	4,114,632	260,854	65.74%
	I		*B. elkanii*	6,907	118,911	8,806	251	1,166	0.18%
	II		*B. elkanii*	6,872	89,370	17,570	631	1,613	0.54%
*Maize + Fusarium:*									
CO354	I	Maize 1^st^	*Z. mays*	257	46,053	63,605,211	5,020,302	1,973,035	7.11%
	II		*Z. mays*	280	43,537	64,060,395	5,074,624	2,015,545	7.13%
	III		*Z. mays*	279	36,277	55,320,528	4,407,818	1,690,684	7.17%
	I		*F. verticillioides*	47	509	2,620,154	218,485	150,957	7.31%
	II		*F. verticillioides*	23	293	1,424,365	119,595	83,640	7.35%
	III		*F. verticillioides*	53	526	3,381,869	282,827	196,770	7.32%
CO354	I	*Fusarium* 1^st^	*Z. mays*	257	35,577	63,061,802	4,983,688	1,678,420	7.14%
	II		*Z. mays*	280	35,190	63,559,272	5,042,606	1,713,630	7.17%
	III		*Z. mays*	279	26,101	54,799,352	4,372,544	1,446,308	7.21%
	I		*F. verticillioides*	48	583	3,139,676	276,241	458,718	7.13%
	II		*F. verticillioides*	23	369	1,903,321	170,812	396,794	6.91%
	III		*F. verticillioides*	53	601	3,879,192	339,538	453,663	7.27%
CO354	I	Combined Analysis	*Z. mays*	257	38,312	63,519,081	5,007,715	1,960,084	7.10%
	II	*Z. mays*	280	37,882	63,994,094	5,066,322	2,006,113	7.13%
	III		*Z. mays*	279	28,336	55,232,851	4,393,751	1,677,822	7.16%
	I		*F. verticillioides*	48	516	2,629,064	221,907	166,912	7.35%
	II		*F. verticillioides*	23	291	1,417,789	121,161	94,107	7.42%
	III		*F. verticillioides*	53	533	3,403,439	287,282	213,560	7.36%

## Discussion

RNA sequencing methodologies are revolutionizing the way we study gene expression. Unlike microarrays, to perform an RNA-Seq analysis there is no need for previous knowledge about the organism. Another advantage of RNA-Seq is that it enables global gene expression analysis since it allows access to different populations of RNA sequences from the organism (Wang *et al.*, 2009, Oshlack *et al.*, 2010). In the last decade, this technique was used to assess gene expression of many organisms and it has recently started to be used to assess the transcriptomes of interacting organisms, called Dual RNA-Seq (Camilios-Neto *et al.*, 2014, Hayden *et al.*, 2014, Baddal *et al.*, 2015, Pankievicz *et al.*, 2016, Westermann *et al.*, 2016).

Despite the difficulties in obtaining libraries containing RNAs from both interacting organisms, there are also problems in sorting the reads *in silico*. The sequential approach seems to be the most common mapping method chosen, and the order of the genomes used in the analysis is chosen according to study interests (Camilios-Neto *et al.*, 2014, Baddal *et al.*, 2015, LaMonte *et al.*, 2019, Mateus *et al.*, 2019, Montoya *et al.*, 2019). Sometimes the reads of one of the interacting organisms are not considered for the study and are discarded from the analysis (Lanubile *et al.*, 2014, Packard *et al.*, 2017, Verwaaijen *et al.*, 2017). Similarly, reads that aligned equally well to either genome or simply cross-mapped are also sometimes discarded (Baddal *et al.*, 2015, Westermann *et al.*, 2016, Westermann and Vogel, 2018).

Here we used a Combined Analysis, which consists in using a Combined Reference file formed by merging the reference genomes files of both organisms to *in silico* sort the reads that align to each genome. Once identified, they were extracted and saved in separated files (Figure S3). The libraries formed by the reads of each organism were then counted using the corresponding reference genome with their own annotations. To perform these analyses, we used the CLC’s tools set with the parameters usually used to map eukaryotic libraries (Camilios-Neto *et al.*, 2014).

Before testing the combined approach, we determined the number of cross-mapped reads between the two RNA-Seq libraries using the reference genome of the other organism of the Combined Reference file. After aligning them, the reads that mapped to the incorrect genome (cross-mapped reads) were counted using both the correct and incorrect reference genome. This was done to identify the loci where these reads were aligned in the incorrect genome and the loci where they should be assigned in the correct one (cross-mapping; Tables 3, S7 and S8). Our results showed that the combined analysis consistently assigned a lower number of reads to the incorrect organism due to cross-map, allowing the program to better attribute the reads to its corresponding genome, leading to a lower number of cross-mappings (Table 1 and S2).

After these cross-map evaluations, two sequential analyses were performed, and the obtained results were compared with the results from the combined analysis. For both sequential analyses, it was possible to notice that the first genome used on the mapping step was always benefited. We observed that the first genome used to map the reads received the full number of reads that could cross-map with the genome of the other organism (Table 1 and S2). We also noticed that even though many of the cross-mapping reads mapped to rRNA genes, a significant number of cross-mapping reads were attributed to CDS loci in all methodological approaches. However, in the combined analysis, the loss of reads due to cross-mapping was lower than in the sequential analysis (Table 3 and S4). Also interesting was the fact that the *H. seropedicae* genome lost more reads for the *Z. mays* genome due to cross-mapping than the other way around.

The sensitivity, specificity, precision, and accuracy of the methodologies in the different parameters tested were also calculated (Tables 1 and S2). According to our results, regardless of the mapping parameters chosen, the Sequential Analysis – Eukaryote 1st presented the worst results for accuracy and precision. On the other hand, the Sequential Analysis – Prokaryote 1st and the Combined analysis presented equivalent results for accuracy and precision, and a slight increase was achieved with more restrictive mapping parameters (Table S2). As the accuracy and precision of the mapping in the Sequential Analysis directly depends on which organism is first used in the analysis, and as the Combined Analysis presented similar values of accuracy and precision as the Sequential Analysis – Prokaryote 1st, we recommend the use of the Combined Analysis since it avoids the tendency of choosing which genome will be the first to be used in the analysis.

To compare the *in silico* data with real Dual RNA-Seq samples, libraries from two different Dual RNA-Seq experiments were submitted to both sequential and the combined approaches. In both experiments, the results obtained were similar and followed the results from the *in silico* data, with the combined analysis showing intermediary values when compared to values attributed by the sequential analyses (Tables S7 and S8). For the *G. max - B. elkanii* experiment, the average amount of reads attributed to the combined analysis was significantly different only concerning the Sequential – Prokaryote 1^st^ data (Figure 2B). Schurch *et al.* (2016) recommended that at least three biological replicates must be used in order to detect genes being differentially expressed. Since the *G. max - B. elkanii* experiment contained only two biological replicates we hypothesized that with more biological replicates these two methodologies should present significant differences concerning the number of reads attributed to each organism.

Another interesting fact was observed in the Lanubile *et al.* (2014) experiment. When comparing the average amount of reads attributed to each genome, regardless of the methodology used, no significant differences were observed (Figure 3). Analyzing our results, it seems that paired-end sequencing was also useful to make the two eukaryotic genomes more distinguishable and less prone to cross-mappings (Figure 3). Therefore, one should consider using paired-end libraries allied with the combined analysis in order to reduce the number of cross-mappings during Dual RNA-Seq experiments.

Since we detected that a significant number of cross-mapping reads aligned to gene coding regions of the genomes, we can assume that this happened because the interacting organisms should have similar metabolic pathways or due to homologous sequences. Eliminating these reads from the libraries before counting them represents a problem because a considerable amount of transcriptional information will be lost. Therefore, all reads that align to both genomes (with different degrees of similarity to each genome) will align to the first genome used in the sequential mapping approach. This might lead to an overestimation of the expressed genes of the first genome used in the sequential mapping method. Similarly, the expressed genes of the second genome might be underestimated. This problem seems to be more critical for those interested in the prokaryotic transcriptome. Prokaryotic RNA is always less abundant in libraries prepared from mixed sources (Westermann *et al.*, 2012); therefore, techniques that underestimate their read counts should be avoided. The combined analysis seems to be more reasonable to avoid these under/overestimations.

Aprianto *et al.* (2016) suggested a Dual RNA-Seq approach in which they aligned the libraries to a chimeric genome. To create this genome, they concatenated the *Streptococcus pneumoniae* genome as an extra chromosome of *Homo sapiens* and adjusted the annotated genomes. All procedures were performed with command-line entries that demands some bioinformatic knowledge and programming skills. Another objective of our work was to describe a way to analyze the Dual RNA-Seq libraries without the need for high computational skills. Therefore, to perform the proposed combined analysis, the CLC Workbench was used. This program is user-friendly since it works with a graphic interface and has several internal tutorials, which demands only basic bioinformatics skills. Another aspect, and according to Baruzzo *et al.* (2017), CLC Workbench, alongside with Novoalign and STAR, is one of the best aligners for eukaryotes in use nowadays, even when using the standard or improved setups.

A critical step during a Dual RNA-Seq experiment is to separate *in silico* the reads that align to each genome. Another reason to use CLC Genomics Workbench is that after performing the mapping step, the program results in a file containing a list showing in which particular reference the reads are aligned. Based on this list, during a combined analysis, the researcher can easily select and extract all the reads that aligned to each reference genome and save them into separate files (Figure S3). As these files will only contain the reads of one organism, the counting step can be performed using the reference genome and annotations of the corresponding organism.

As a conclusion, with the present work we were able to show that Dual RNA-Seq results vary according to the mapping strategy chosen and this could lead to misinterpretations of the interactions between organisms. Our results showed that the combined analysis allows a smaller loss of reads due to cross-mapping. This fact avoids the loss of relevant information to the first genome chosen in the mapping step when the sequential analysis is used. Since most studies first align the RNA-Seq libraries to the eukaryotic genome, much prokaryotic information is probably being lost. Thus, to fully comprehend gene expression and communication between interacting organisms, we suggest adopting the combined mapping analysis in Dual RNA-Seq experiments.

## Supplementary material

The following online material is available for this article:

Figure S1 Filtering procedure and construction of the Chimera library.

Figure S2 Mapping strategies to determine the number of cross-mapping reads.

Figure S3 Step-by-step of how to extract the reads after the mapping step using the Combined Reference in the CLC workbench environment.

Table S1 Matrix indicating the reads from (A) *Z. mays* or (B) *Herbaspirillum* that were used to determine the true positive (TP), true negative (TN), false positive (FP) and false negative (FN).

Table S2 Library features and number of total reads attributed to the *Herbaspirillum seropedicae* or *Zea mays* genomes according to the mapping approach.

Table S3 Comparison of the number of reads and where these reads were mapped in each reference genome with the mapping parameters of 0.9 of minimum length fraction and 0.8 of minimum similarity fraction.

Table S4 Comparison of the number of reads incorrectly mapped due to cross-mapping, with the mapping parameters of 0.9 of minimum length fraction and 0.8 of minimum similarity fraction.

Table S5 Top 20 most counted loci whose reads were lost from the *Herbaspirillum* (A) and *Z. mays* (B) libraries.

Table S6 Top 20 most counted loci whose reads were gained to the *Herbaspirillum* (A) and *Z. mays* (B) libraries.

Table S7 Library features and number of total reads attributed to the *Bradyrhizobium elkanii* or *Glycine max* genomes according to the mapping approach.

Table S8 Library features and number of total reads attributed to the *Fusarium verticillioides* or *Zea mays* genomes according to the mapping approach.
