# Supplementary material for: The combined analysis as the best strategy for Dual RNA-Seq mapping
Source: Genet Mol Biol. 2020 Feb 10;42(4):e20190215. doi: 10.1590/1678-4685-GMB-2019-0215 (PMC7249662; doi:10.1590/1678-4685-GMB-2019-0215)
Supplement: Supplementary file 1 [file 1415-4757-GMB-42-4-e20190215-s1.pdf]

Supplementary Material to "The combined analysis as the best strategy for Dual RNA-Seq mapping"

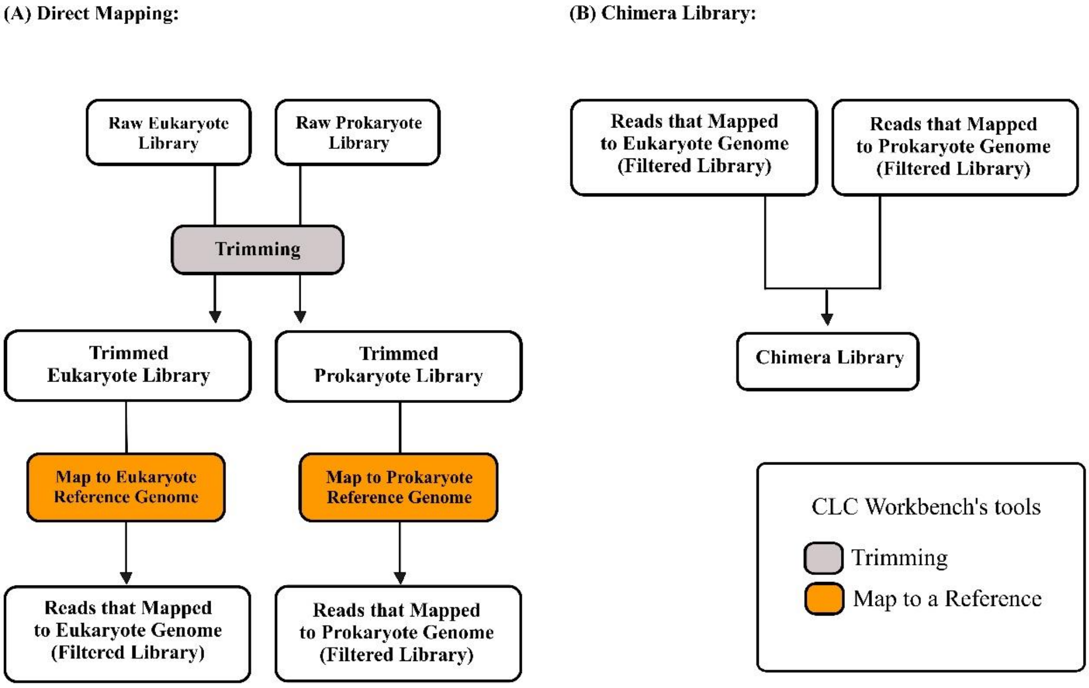

Figure S1 - Filtering procedure and construction of the Chimera library. (A) Direct mapping used to filter the libraries to eliminate potential contamination reads (those that do not map to the respective genome). (B) Construction of the Chimera Library by merging the files with reads that mapped to the *Herbaspirillum seropedicae* and *Zea mays* reference genomes only.
