# Supplementary material for: The combined analysis as the best strategy for Dual RNA-Seq mapping
Source: Genet Mol Biol. 2020 Feb 10;42(4):e20190215. doi: 10.1590/1678-4685-GMB-2019-0215 (PMC7249662; doi:10.1590/1678-4685-GMB-2019-0215)
Supplement: Supplementary file 2 [file 1415-4757-GMB-42-4-e20190215-s2.pdf]

Supplementary Material to "The combined analysis as the best strategy for Dual RNA-Seq mapping"

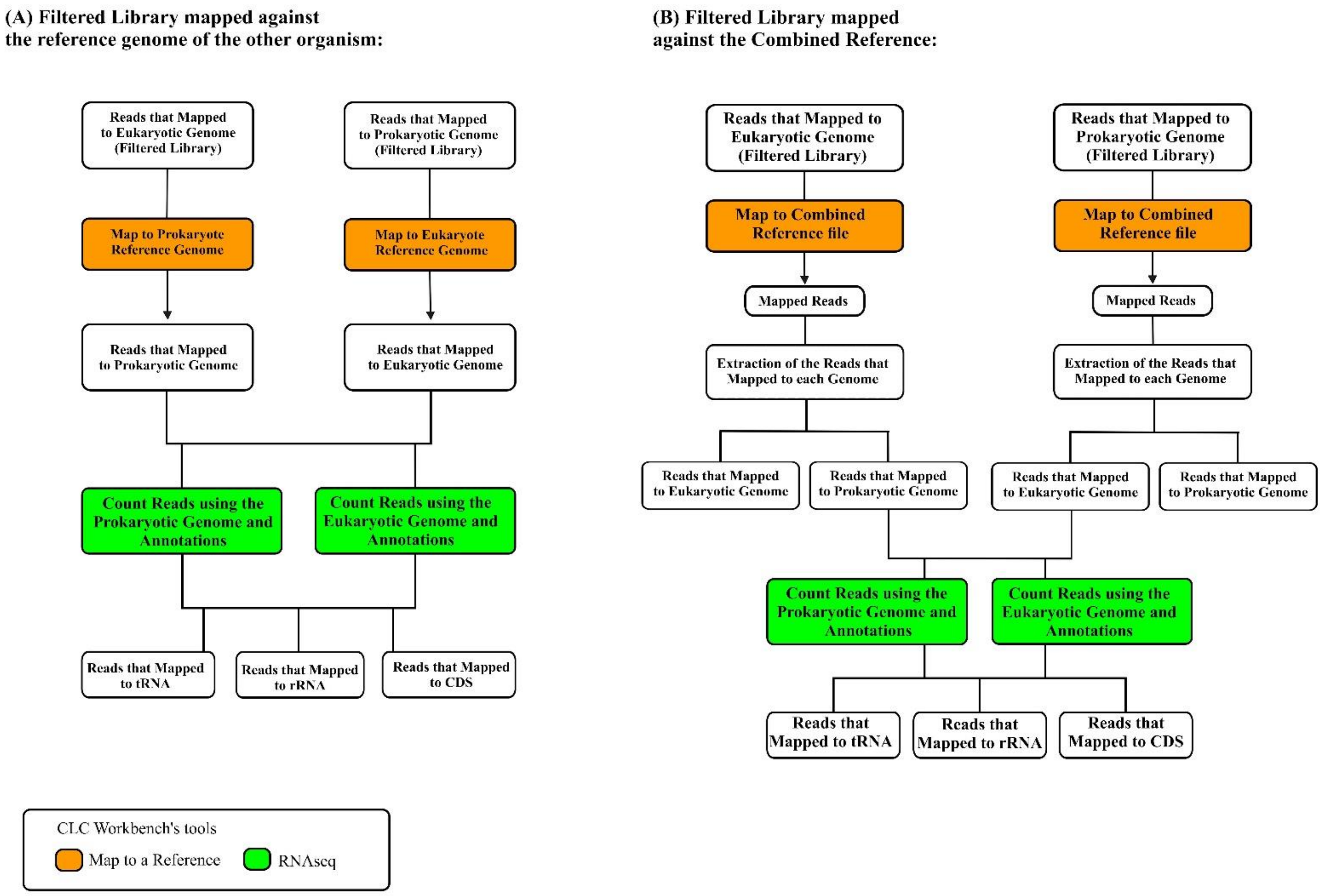

Figure S2 - Mapping strategies to determine the number of cross-mapping reads. (A) Filtered libraries mapped to the reference genome of the other organism. (B) Filtered libraries mapped to the Combined Genomes.
