# Supplementary material for: The combined analysis as the best strategy for Dual RNA-Seq mapping
Source: Genet Mol Biol. 2020 Feb 10;42(4):e20190215. doi: 10.1590/1678-4685-GMB-2019-0215 (PMC7249662; doi:10.1590/1678-4685-GMB-2019-0215)
Supplement: Supplementary file 3 [file 1415-4757-GMB-42-4-e20190215-s4.pdf]

Supplementary Material to “The combined analysis as the best strategy for Dual RNA-Seq mapping”

Table S1 - Matrix indicating the reads from (A) *Z. mays* or (B) *Herbaspirillum* that were used to determine the true positive (TP), true negative (TN), false positive (FP) and false negative (FN). These data were used to calculate the sensitivity, specificity, accuracy and precision of each methodology using the formulas presented in (C).

(A) *Z. mays*

		Classification	
		Maize	Non-maize
Reads	Maize	Maize reads classified as **belonging** to maize (TP)	Maize reads classified as **not belonging** to maize (FN)
	Non-maize	Bacterial reads classified as **belonging** to maize (FP)	Bacterial reads classified as **not belonging** to maize (TN)

(B) *Herbaspirillum*

		Classification	
		Bacterium	Non-Bacterium
Reads	Bacterium	Bacterial reads classified as **belonging** to the bacteria (TP)	Bacterial reads classified as **not belonging** to the bacteria (FN)
	Non-Bacterium	Maize reads classified as **belonging** to the bacteria (FP)	Maize reads classified as **not belonging** to the bacteria (TN)

(C)

Parameter	Sensitivity (True Positive Rate – TPR)	Specificity (True Negative Rate – TNR)	Accuracy (ACC)	Precision (P)
Formula	$TPR = \frac{TP}{(TP + FN)}$	$TNR = \frac{TN}{(TN + FP)}$	$ACC = \frac{(TP + TN)}{(TP + TN + FP + FN)}$	$P = \frac{TP}{(TP + FP)}$
