# Supplementary material for: The combined analysis as the best strategy for Dual RNA-Seq mapping
Source: Genet Mol Biol. 2020 Feb 10;42(4):e20190215. doi: 10.1590/1678-4685-GMB-2019-0215 (PMC7249662; doi:10.1590/1678-4685-GMB-2019-0215)
Supplement: Supplementary file 4 [file 1415-4757-GMB-42-4-e20190215-s3.pdf]

Supplementary Material to "The combined analysis as the best strategy for Dual RNA-Seq mapping"

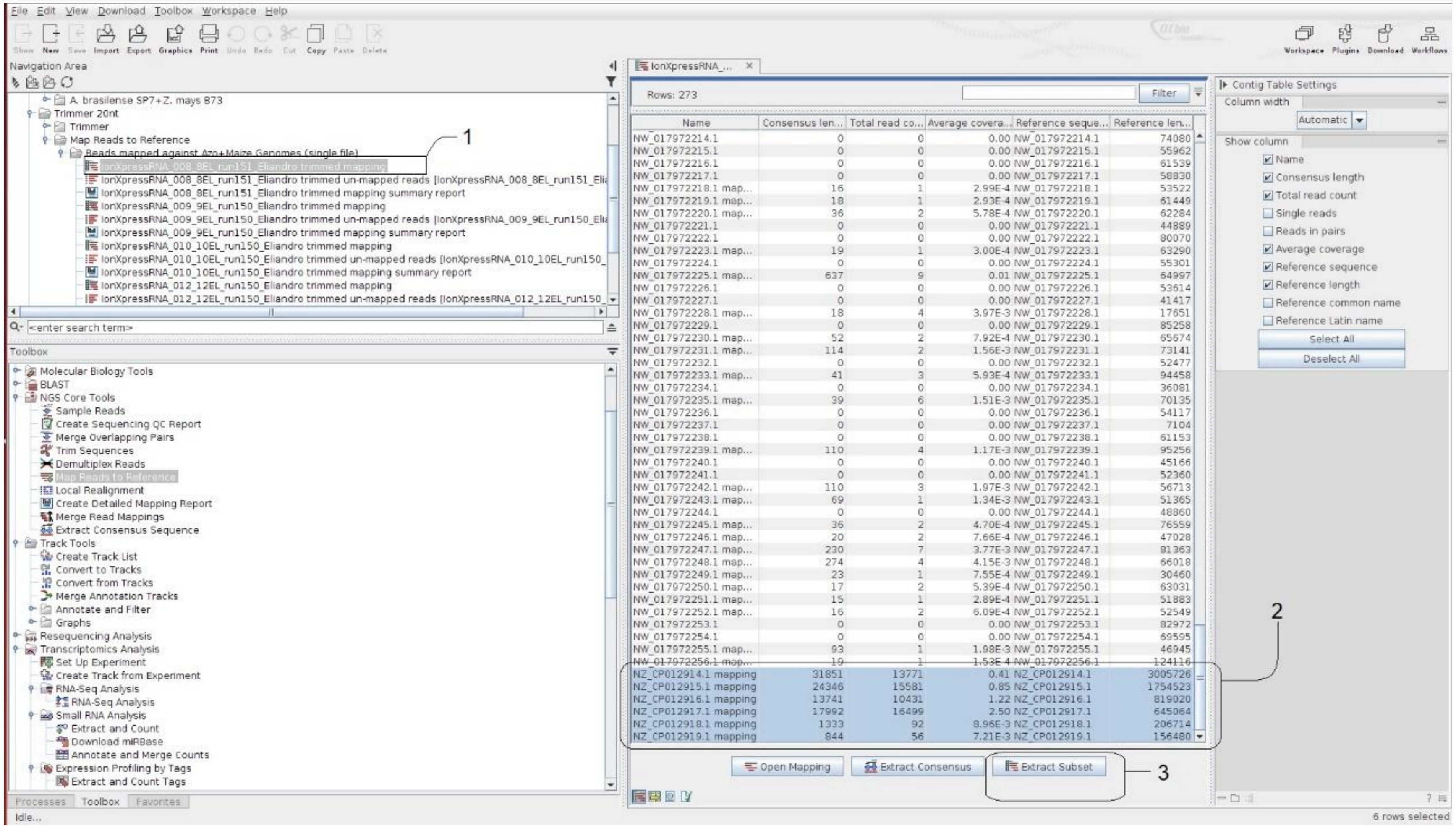

Figure S3 Step-by-step of how to extract the reads after the mapping step using the Combined Reference in the CLC workbench environment. 1. Select the result file; 2. Select the lines with the reads of the organism; 3. Click on "Extract Subset". In the following window, select a place to save and the new file will contain all the reads that mapped to that genome, without the need of any extra alignment step.
