# Supplementary material for: The combined analysis as the best strategy for Dual RNA-Seq mapping
Source: Genet Mol Biol. 2020 Feb 10;42(4):e20190215. doi: 10.1590/1678-4685-GMB-2019-0215 (PMC7249662; doi:10.1590/1678-4685-GMB-2019-0215)
Supplement: Supplementary file 5 [file 1415-4757-GMB-42-4-e20190215-s5.pdf]

Supplementary Material to "The combined analysis as the best strategy for Dual RNA-Seq mapping"

Table S2 - Library features and number of total reads attributed to the *Herbaspirillum seropedicae* or *Zea mays* genomes according to the mapping approach. The analyses were performed with the genomes without annotations, with the mapping parameters of 0.9 of minimum length fraction and 0.8 of minimum similarity fraction. Values for sensitivity, specificity, accuracy, and precision were determined according to Table S1.

Library	Total Reads	Total reads after trimming	Number of Reads After Library filtration	Cross-Mapping[1]		Mapping Strategy						
						Sequential Analysis[2]				Combined Analysis[3]		
						Eukaryote 1st		Prokaryote 1st				
				H. seropedicae	*Z. mays*	*H. seropedicae*	*Z. mays*	*H. seropedicae*	*Z. mays*	*H. seropedicae*	*Z. mays*	unmapped
H. seropedicae	158,053,843	92,987,843	41,331,508	-	8,965,305	-	-	-	-	40,781,570	505,738	44,200
Z. mays	24,300,211	24,255,170	21,415,293	4,341	-	-	-	-	-	267	21,415,003	23
Chimera Library	-	-	62,746,801	-	-	32,366,203	30,380,598	41,335,849	21,410,952	40,781,843	21,920,735	44,223
Sensitivity	-	-	-	-	-	0.7735	1.0000	1.0000	0.9998	0.9825	1.0000	-
Specificity	-	-	-	-	-	1.0000	0.7831	0.9998	1.0000	1.0000	0.9878	-
Accuracy	-	-	-	-	-	0.8530	0.8571	0.9999	0.9999	0.9883	0.9919	-
Precision	-	-	-	-	-	1.0000	0.7049	0.9999	1.0000	1.0000	0.9769	-

[1]Cross-mapping: reads of each individual library were mapped to the reference genome of the other organism. [2]Sequential Analysis: The library was first mapped to one reference genome, reads that fail to map to the first genome were mapped to the other genome. Eukaryote 1st/Prokaryote 1st indicates the first reference used. [3]Combined analysis: libraries were mapped to a merged file containing both reference genomes (Combined Reference).
