# Supplementary material for: The combined analysis as the best strategy for Dual RNA-Seq mapping
Source: Genet Mol Biol. 2020 Feb 10;42(4):e20190215. doi: 10.1590/1678-4685-GMB-2019-0215 (PMC7249662; doi:10.1590/1678-4685-GMB-2019-0215)
Supplement: Supplementary file 6 [file 1415-4757-GMB-42-4-e20190215-s6.pdf]

Supplementary Material to “The combined analysis as the best strategy for Dual RNA-Seq mapping”

Table S3 - Comparison of the number of reads and where these reads were mapped in each reference genome according to the methodology used, with the mapping parameters of 0.9 of minimum length fraction and 0.8 of minimum similarity fraction. The unmapped reads are due to one of the counting parameters that eliminate reads that mapped in more than 5 loci.

Mapping Strategy	Library	Reference Used to Count the Reads	Number of Reads Mapped to				Unmapped reads	Proportion of Multireads from total
			tRNA	rRNA	CDS loci			
					Unique	Multi		
Direct Mapping	*H. seropedicae*	*H. seropedicae*	1,051,140	30,025,293	8,074,476	61,241	2,119,358	0.15%
	Z. mays	*Z. mays*	843	2,578	19,329,900	800,746	1,281,226	3.74%
Eukaryote 1st	Chimera Library	*H. seropedicae*	909,567	23,196,969	6,385,015	34,426	1,840,226	0.11%
		Z. mays	46,847	2,714,236	21,966,803	1,692,284	3,960,428	5.57%
Prokaryote 1st	Chimera Library	*H. seropedicae*	1,051,141	30,025,885	8,077,444	61,595	2,119,784	0.15%
		Z. mays	840	2,012	19,326,915	800,137	1,281,048	3.74%
Combined Analysis	Chimera Library	*H. seropedicae*	1,049,225	29,799,822	7,779,782	51,260	2,101,754	0.13%
		Z. mays	1,020	70,836	19,573,804	859,314	1,415,761	3.92%
