# Supplementary material for: The combined analysis as the best strategy for Dual RNA-Seq mapping
Source: Genet Mol Biol. 2020 Feb 10;42(4):e20190215. doi: 10.1590/1678-4685-GMB-2019-0215 (PMC7249662; doi:10.1590/1678-4685-GMB-2019-0215)
Supplement: Supplementary file 7 [file 1415-4757-GMB-42-4-e20190215-s7.pdf]

Supplementary Material to "The combined analysis as the best strategy for Dual RNA-Seq mapping"

Table S4 - Comparison of the number of reads incorrectly mapped due to cross-mapping, with the mapping parameters of 0.9 of minimum length fraction and 0.8 of minimum similarity fraction. Reads that incorrectly mapped to the reference genome were counted using the annotated genome indicated on the table. The unmapped reads are a result of the counting parameters that eliminate reads that mapped in more than five loci and in the intergenic regions.

Library	Reference Used to Map the Reads	Reference Used to Count the Cross-Mapped Reads	Number of Reads Mapped to			CDS*	Unmapped reads
			tRNA	rRNA	CDS Loci		
H. seropedicae	*Z. mays*	*H. seropedicae*	141,573	6,828,324	1,716,276	4,288	279,132
		Z. mays	46,004	2,711,658	3,528,441	16,736	2,679,202
	Combined Reference	*H. seropedicae*	1,690	220,341	267,338	1,844	16,369
		Z. mays	177	68,472	302,524	1,426	134,565
Z. mays	*H. seropedicae*	*Z. mays*	3	566	3,594	35	178
		H. seropedicae	1	592	3,322	29	426
	Combined Reference	*Z. mays*	0	200	41	29	26
		H. seropedicae	0	220	42	33	5

*CDS with at least 10 reads assigned to them. Exception made to the *Z. mays* library mapped against the Combined Reference, which refers to CDS with at least one read assigned to it.
