# Supplementary material for: The combined analysis as the best strategy for Dual RNA-Seq mapping
Source: Genet Mol Biol. 2020 Feb 10;42(4):e20190215. doi: 10.1590/1678-4685-GMB-2019-0215 (PMC7249662; doi:10.1590/1678-4685-GMB-2019-0215)
Supplement: Supplementary file 8 [file 1415-4757-GMB-42-4-e20190215-s8.pdf]

Supplementary Material to "The combined analysis as the best strategy for Dual RNA-Seq mapping"

Table S5 - Top 20 most counted loci whose reads were lost from the *Herbaspirillum* (A) and *Z. mays* (B) libraries according to the mapping strategy used, with the mapping parameters of 0.8 of minimum length fraction and 0.8 of minimum similarity fraction.

(A)

Mapping Strategy	Name	Description	RPKM	Unique gene reads	Total gene reads
Cross-Mapping	ACP92_RS13610	hypothetical protein	252,245	257,953	258,832
	ssrA_2	miscRNA (Gene Ids: 31910439 and 31910466)	98,920	112,858	113,078
	ACP92_RS17815	glycosyltransferase	17,899	97,268	97,311
	ACP92_RS21415	porin	20,850	72,152	72,816
	ACP92_RS17420	NADPH:quinone reductase	16,426	50,629	50,713
	ACP92_RS18115	nitrate reductase subunit alpha	3,998	44,417	44,736
	ACP92_RS12550	ethanolamine ammonia lyase large subunit	7,459	31,460	31,489
	ACP92_RS19205	amino acid ABC transporter substrate-binding protein	10,559	29,880	30,033
	ACP92_RS01180	acetyl-CoA acetyltransferase	8,266	29,044	29,164
	ACP92_RS14850	2-oxoglutarate dehydrogenase subunit E1	3,142	26,952	27,010
	ACP92_RS18120	nitrate reductase subunit beta	4,957	22,997	23,112
	ACP92_RS19810	ribosomal subunit interface protein	18,490	20,061	20,138
	ACP92_RS14915	chemotaxis protein CheW	11,625	17,680	17,684
	ACP92_RS14905	aconitate hydratase	2,140	17,291	17,417
	ACP92_RS06995	ATP-dependent Clp protease ATP-binding subunit ClpA	2,392	16,485	16,536
	ACP92_RS19805	RNA polymerase sigma-54 factor	3,653	16,323	16,340
	ACP92_RS12875	endopeptidase La	2,246	16,181	16,231
	ACP92_RS18395	membrane protein	8,387	15,375	15,400

	ACP92_RS23935	ABC transporter permease	4,182	14,906	15,169
	ACP92_RS10320	flagellin	4,123	13,438	13,472
Combined	ACP92_RS13610	hypothetical protein	804,058	113,238	113,796
	ACP92_RS17815	glycosyltransferase	128,865	96,587	96,629
	ACP92_RS15580	alkene reductase	9,763	4,373	4,424
	ACP92_RS14850	2-oxoglutarate dehydrogenase subunit E1	2,792	3,267	3,310
	ACP92_RS12550	ethanolamine ammonia lyase large subunit	3,674	2,114	2,139
	ssrA_2	miscRNA (Gene Ids: 31910439 and 31910466)	10,294	1,438	1,623
	ACP92_RS11435	hypothetical protein	12,683	1,542	1,543
	ACP92_RS07545	ribosome maturation factor RimM	6,000	1,522	1,527
	ACP92_RS01180	acetyl-CoA acetyltransferase	3,136	1,439	1,526
	ACP92_RS23050	hypothetical protein	4,017	1,415	1,491
	ACP92_RS18115	nitrate reductase subunit alpha	844	1,173	1,302
	ACP92_RS20135	hypothetical protein	4,981	1,243	1,243
	ACP92_RS17420	NADPH:quinone reductase	2,907	1,185	1,238
	ACP92_RS07625	RND transporter	1,830	1,088	1,154
	ACP92_RS05425	hypothetical protein	116	828	1,143
	ACP92_RS21415	porin	2,371	1,077	1,142
	ACP92_RS06190	protein translocase subunit SecD	1,444	1,068	1,138
	ACP92_RS20600	phenylacetic acid degradation protein PaaD	5,563	616	1,098
	ACP92_RS18120	nitrate reductase subunit beta	1,690	1,032	1,087
	ACP92_RS18395	membrane protein	4,079	1,013	1,033

(B)

	Name	Description	RPKM	Unique gene reads	Total gene reads
Cross-Mapping	LOC103629174	trihelix transcription factor GT-3b	8,504	114	279
	LOC542551	60S acidic ribosomal protein P2A-like	20,418	269	277
	LOC542323	acidic ribosomal protein P2a (ARPP2A)	18,561	260	263
	pco106379	RNA-binding (RRM/RBD/RNP motifs) family protein	15,534	182	208
	LOC542725	uncharacterized LOC542725	33,925	192	203
	LOC542337	acidic ribosomal protein P2a-2 (ARPP2A-2)	12,514	165	166
	LOC103627451	ribosomal RNA-processing protein 14	15,394	125	147
	fdh1	formaldehyde dehydrogenase homolog 1 (fdh1)	5,476	131	131
	LOC100194217	responsive to abscisic acid15	21,139	115	124
	pco102485	uncharacterized LOC100282592	2,842	90	93
	LOC103638017	ethylene-responsive transcription factor RAP2-1	9,638	60	77
	LOC100286241	heat shock 70 kDa protein	3,667	71	71
	LOC100194313	acidic ribosomal protein P2a-4	5,246	68	71
	HMG13	high mobility group protein 1	4,573	63	63
	LOC100193124	myosin-like protein	1,537	58	58
	gpm583	Guanine nucleotide-binding protein beta subunit-like protein	3,351	49	49
	LOC103639924	probable mediator of RNA polymerase II transcription subunit 37c	1,852	44	47
	LOC100501446	uncharacterized LOC100501446	12,593	0	43
	LOC109939197	NADP-dependent malic enzyme, chloroplastic	1,132	0	41
	LOC103650526	probable mediator of RNA polymerase II transcription subunit 37c	1,534	36	40
Combined	pco106379	RNA-binding (RRM/RBD/RNP motifs) family protein	42,937	1	5
	LOC103648227	AAA-ATPase ASD mitochondrial	15,256	1	2
	LOC109944797	cold and drought-regulated protein CORA	34,983	0	2

	LOC109940833	NAD(P)H-quinone oxidoreductase subunit 5, chloroplastic-like	3,499	0	2
	LOC103632470	aminotransferase ALD1 homolog	3,736	1	1
	LOC103634426	probable protein phosphatase 2C 31	5,291	1	1
	LOC100502427	uncharacterized LOC100502427	1,606	1	1
	umc2536	uncharacterized LOC100275320	20,689	0	1
	LOC100281029	GIR1	22,873	1	1
	LOC103650526	probable mediator of RNA polymerase II transcription subunit 37c	4,410	1	1
	LOC100272685	uncharacterized LOC100272685	6,075	1	1
	LOC100194009	uncharacterized LOC100194009	1,085	1	1
	LOC100191812	uncharacterized LOC100191812	1,003	1	1
	LOC103651416	DUF538 family protein	24,265	0	1
	LOC103655071	probable pectinesterase/pectinesterase inhibitor 46	34,000	0	1
	LOC100275424	uncharacterized LOC100275424	7,843	0	1
	LOC100275783	uncharacterized LOC100275783	5,362	0	1
	LOC542333	cytosolic glyceroldehyde-3-phosphate dehydrogenase (GAPC3)	5,438	0	1
	LOC100191329	uncharacterized LOC100191329	4,888	0	1
	LOC100279325	meiotic recombination protein SPO11	2,499	1	1

Unique gene reads = reads that mapped on a single locus; Total gene reads= sum of unique gene reads and reads that mapped to more than 5 loci; RPKM = reads per kilobase million.
