# Supplementary material for: The combined analysis as the best strategy for Dual RNA-Seq mapping
Source: Genet Mol Biol. 2020 Feb 10;42(4):e20190215. doi: 10.1590/1678-4685-GMB-2019-0215 (PMC7249662; doi:10.1590/1678-4685-GMB-2019-0215)
Supplement: Supplementary file 9 [file 1415-4757-GMB-42-4-e20190215-s9.pdf]

Supplementary Material to “The combined analysis as the best strategy for Dual RNA-Seq mapping”

Table S6 - Top 20 most counted loci whose reads were gained to the *Herbaspirillum* (A) and *Z. mays* (B) libraries according to the mapping strategy used, with the mapping parameters of 0.8 of minimum length fraction and 0.8 of minimum similarity fraction.

(A)

Mapping Strategy	Name	Description	RPKM	Unique gene reads	Total gene reads
Cross-Mapping	ACP92_RS15225	cell wall surface anchor protein	2,544	333	339
	ACP92_RS16690	ABC transporter substrate-binding protein	36,149	228	298
	ACP92_RS07965	heavy metal translocating P-type ATPase	19,151	232	277
	ACP92_RS19745	single-stranded DNA-binding protein	42,097	133	136
	ACP92_RS01655	UDP-N-acetylmuramoyl-L-alanine--D-glutamate ligase	9,772	116	129
	ACP92_RS13570	S-(hydroxymethyl)glutathione dehydrogenase	18,829	129	129
	ACP92_RS04965	molecular chaperone GroEL	12,339	126	126
	ACP92_RS23590	Ni/Fe hydrogenase	30,098	96	114
	ACP92_RS01565	MexE family multidrug efflux RND transporter periplasmic adaptor subunit	13,195	93	98
	ACP92_RS10470	phosphatidylinositol kinase	11,649	72	93
	ACP92_RS18805	two-component sensor histidine kinase	4,412	77	77
	ACP92_RS05425	hypothetical protein	477	68	70
	ACP92_RS16420	colicin V production protein	21,931	65	68
	ACP92_RS00385	serine/threonine protein phosphatase	5,105	60	60
	ACP92_RS07325	hypothetical protein	12,673	60	60
	ACP92_RS03935	EscV/YscV/HrcV family type III secretion system export apparatus protein	3,792	42	49
	ACP92_RS12400	glyoxylate/hydroxypyruvate reductase A	7,911	26	47
	ACP92_RS17960	23S rRNA pseudouridylate synthase B	3,506	44	44

	ACP92_RS17105	RNA helicase	4,216	37	40
	ACP92_RS13260	membrane protein	3,993	39	39
Combined	ACP92_RS16605	hypothetical protein	313,873	5	5
	ACP92_RS19745	single-stranded DNA-binding protein	129,879	4	4
	ACP92_RS05425	hypothetical protein	1,428	2	2
	ACP92_RS07830	FAD-dependent oxidoreductase	22,735	2	2
	ACP92_RS20885	hypothetical protein	61,746	2	2
	ACP92_RS00010	DNA polymerase III subunit beta	15,311	1	1
	ACP92_RS00220	hypothetical protein	41,850	1	1
	ACP92_RS00520	DNA-directed RNA polymerase subunit beta	4,127	1	1
	ACP92_RS00925	hypothetical protein	10,992	1	1
	ACP92_RS24015	hypothetical protein	19,152	1	1
	ACP92_RS03200	IMP dehydrogenase	5,004	1	1
	ACP92_RS03310	hypothetical protein	27,831	1	1
	ACP92_RS03695	hypothetical protein	26,903	1	1
	ACP92_RS04410	phage late control protein	13,780	1	1
	ACP92_RS04995	transcriptional regulator	18,225	1	1
	ACP92_RS05310	ribulokinase	10,291	1	1
	ACP92_RS05790	IclR family transcriptional regulator	21,400	1	1
	ACP92_RS05930	branched-chain amino acid ABC transporter permease	18,225	0	1
	ACP92_RS06930	DNA-binding response regulator	24,458	1	1
	ACP92_RS07090	type I glyceraldehyde-3-phosphate dehydrogenase	16,715	1	1

(B)

Mapping Strategy	Name	Description	RPKM	Unique gene reads	Total gene reads
Cross-Mapping	LOC103641534	Protein RAE1	6,058	704,808	820,795
	LOC542347	thylakoid assembly 1	2,920	294,791	410,812
	LOC103649599	cationic amino acid transporter 5	18,714	251,214	252,269
	LOC100192995	Senescence-associated protein 5	12,305	158,512	168,863
	LOC100277826	Tubulin binding cofactor C domain-containing protein	1,134	108,260	128,598
	LOC103630900	uncharacterized LOC103630900	707	98,503	126,088
	LOC100283525	uncharacterized LOC100283525	3,918	118,394	124,591
	LOC103643067	endoplasmic reticulum-Golgi intermediate compartment protein 3	2,787	109,149	119,315
	LOC103654984	wall-associated receptor kinase 2	2,240	53,157	86,684
	LOC100381550	uncharacterized LOC100381550	236	403	82,691
	LOC103626304	protein ACTIVITY OF BC1 COMPLEX KINASE 7, chloroplastic	231	9,148	81,110
	LOC100191576	Seed maturation protein	257	59,014	61,028
	LOC103654469	LRR receptor-like serine/threonine-protein kinase	1,870	44,808	47,297
	LOC103629816	cell division cycle and apoptosis regulator protein 1	518	45,032	45,755
	LOC100272896	hypothetical protein	1,176	45,107	45,527
	LOC100285109	uncharacterized LOC100285109	916	37,805	44,497
	LOC100285492	receptor-like protein kinase 5	1,535	34,481	43,216
	LOC103632082	tRNA ligase 1	216	287	41,502
	LOC100216999	uncharacterized LOC100216999	1,760	37,941	38,329
	LOC103626482	callose synthase 3	82	25,228	37,686
Combined	LOC103649599	cationic amino acid transporter 5	97,605	109,238	109,518
	LOC103654984	wall-associated receptor kinase 2	27,725	53,102	89,322
	LOC103641534	Protein RAE1	3,590	3,885	40,486

	LOC542347	thylakoid assembly 1	3,086	33,813	36,145
	LOC100191447	uncharacterized LOC100191447	2,619	4,684	6,142
	LOC100276421	GW2	909	72	2,662
	LOC103626482	callose synthase 3	63	1,716	2,383
	LOC109941752	translation initiation factor IF-2-like	100	269	2,177
	LOC100384061	uncharacterized LOC100384061	2,037	1,677	1,710
	LOC100276350	CASP-like protein 13	95	1,681	1,699
	LOC103636141	mitogen-activated protein kinase 8-like	508	1,672	1,673
	LOC100273234	PLAC8 family protein	1,681	433	1,666
	LOC100282489	uncharacterized LOC100282489	321	1,319	1,570
	LOC100276590	uncharacterized LOC100276590	645	1,499	1,544
	LOC100384715	hypothetical protein	1,512	1,464	1,472
	LOC103637150	uncharacterized LOC100384715	417	1,116	1,411
	LOC103643382	tubulin-folding cofactor D	210	1,133	1,410
	LOC100286182	uncharacterized LOC100286182	550	450	1,352
	LOC103634584	ncRNA	1,545	1,313	1,320
	LOC103626598	uncharacterized LOC103634584	1,056	853	1,229

Unique gene reads = reads that mapped on a single locus; Total gene reads= sum of unique gene reads to reads that mapped to more than 5 loci; RPKM = reads per kilobase million.
