# Supplementary material for: The combined analysis as the best strategy for Dual RNA-Seq mapping
Source: Genet Mol Biol. 2020 Feb 10;42(4):e20190215. doi: 10.1590/1678-4685-GMB-2019-0215 (PMC7249662; doi:10.1590/1678-4685-GMB-2019-0215)
Supplement: Supplementary file 10 [file 1415-4757-GMB-42-4-e20190215-s10.pdf]

Supplementary Material to "The combined analysis as the best strategy for Dual RNA-Seq mapping"

Table S7 - Library features and number of total reads attributed to the *Bradyrhizobium elkanii* or *Glycine max* genomes according to the mapping approach, with the mapping parameters of 0.8 of minimum length fraction and 0.8 of minimum similarity fraction. BR16 and ER48: soybean varieties BR16 and Embrapa 48, respectively.

**Samples**	**Biological Replicate**	**Total Reads**	**Total reads after trimming**	Mapping Strategy								
				Sequential Analysis						Combined Analysis		
				Eukaryote 1st			Prokaryote 1st					
				B. elkanii	*G. max*	Unmapped	*B. elkanii*	*G. max*	Unmapped	*B. elkanii*	*G. max*	Unmapped
BR16	I	9,065,000	9,030,791	163,688	7,634,446	1,232,657	306,239	7,491,895	1,232,657	200,701	7,590,891	1,239,199
	II	9,792,000	9,750,645	185,255	7,909,778	1,655,612	356,332	7,738,701	1,655,612	234,583	7,853,932	1,662,130
ER48	I	5,693,000	5,663,651	102,174	4,725,878	835,599	199,945	4,628,107	835,599	136,041	4,688,890	838,720
	II	7,308,000	7,255,860	88,046	6,289,214	878,600	209,424	6,167,836	878,600	116,056	6,258,515	881,289
