# Supplementary material for: The combined analysis as the best strategy for Dual RNA-Seq mapping
Source: Genet Mol Biol. 2020 Feb 10;42(4):e20190215. doi: 10.1590/1678-4685-GMB-2019-0215 (PMC7249662; doi:10.1590/1678-4685-GMB-2019-0215)
Supplement: Supplementary file 11 [file 1415-4757-GMB-42-4-e20190215-s11.pdf]

# Supplementary Material to "The combined analysis as the best strategy for Dual RNA-Seq mapping"

**Table S8 -** Library features and number of total reads attributed to the *Fusarium verticillioides* or *Zea mays* genomes according to the mapping approach, with the mapping parameters of 0.8 of minimum length fraction and 0.8 of minimum similarity fraction. Reads in Pairs indicates the total number of reads that were aligned in pairs. Broken Pair Reads indicates pair-reads that could be aligned independently, none of the possible placements of the pair satisfies the pairing criteria. CO354: susceptible maize variety CO354 inoculated with *F. verticillioides* from Lanubile *et al.* (2014).

| **Sample** | **Biological Replicate** | **Total reads after trimming** | Sequential Analysis | | | | | | | |
|---|---|---|---|---|---|---|---|---|---|---|
| | | | Maize 1st | | | | *Fusarium* 1st | | | |
| | | | *F. verticillioides* | | *Z. mays* | | *F. verticillioides* | | *Z. mays* | |
| | | | Reads in pairs | Broken paired reads | Reads in pairs | Broken paired reads | Reads in pairs | Broken paired reads | Reads in pairs | Broken paired reads |
| | I | 83,693,936 | 2,638,310 | 351,842 | 57,503,900 | 13,140,958 | 3,073,034 | 802,232 | 55,856,108 | 13,903,636 |
| CO354 | II | 82,742,688 | 1,412,600 | 215,316 | 58,018,836 | 13,175,545 | 1,790,488 | 680,831 | 57,277,376 | 13,073,602 |
| | III | 74,252,520 | 3,422,402 | 439,643 | 50,135,992 | 11,319,594 | 3,887,094 | 785,953 | 49,000,248 | 11,644,336 |

(Continuing Table S8)

| **Sample** | **Biological Replicate** | **Total reads after trimming** | Combined Analysis | | | | |
|---|---|---|---|---|---|---|---|
| | | | *F. verticillioides* | | *Z. mays* | | |
| | | | Reads in pairs | Broken paired reads | Reads in pairs | Broken paired reads | Total Reads |
| | I | 83,693,936 | 2,756,392 | 262,055 | 57,223,066 | 13,302,383 | 73,543,896 |
| CO354 | II | 82,742,688 | 1,468,926 | 164,445 | 57,841,826 | 13,262,865 | 72,738,062 |
| | III | 74,252,520 | 3,604,734 | 300,133 | 50,026,720 | 11,306,319 | 65,237,906 |

**Reference**

Lanubile A, Ferrarini A, Maschietto V, Delledonne M, Marocco A and Bellin D (2014) Functional genomic analysis of constitutive and inducible defense responses to *Fusarium verticillioides* infection in maize genotypes with contrasting ear rot resistance. BMC Genomics 15:710.
